# AcBBX5, a B-box transcription factor from pineapple, regulates flowering time and floral organ development in plants

**DOI:** 10.3389/fpls.2022.1060276

**Published:** 2022-11-24

**Authors:** Yanwei Ouyang, Xiumei Zhang, Yongzan Wei, Yukun He, Xiaohan Zhang, Ziqiong Li, Can Wang, Hongna Zhang

**Affiliations:** ^1^ Key Laboratory for Quality Regulation of Tropical Horticultural Crops of Hainan Province, School of Horticulture, Sanya Nanfan Research Institute, Hainan University, Haikou, China; ^2^ Key Laboratory of Ministry of Agriculture for Tropical Fruit Biology, South Subtropical Crops Research Institute, Chinese Academy of Tropical Agricultural Sciences, Zhanjiang, China; ^3^ Key Laboratory of Biology and Genetic Resources of Tropical Crops, Ministry of Agriculture, Institute of Tropical Bioscience and Biotechnology, Chinese Academy of Tropical Agricultural Sciences, Hainan Institute for Tropical Agricultural Resources, Haikou, China

**Keywords:** pineapple (*Ananas comosus* (L.) Merr.), B-box, expression profiling, flowering, floral organ

## Abstract

Flowering is an important factor to ensure the success of plant reproduction, and reasonable flowering time is crucial to the crop yield. BBX transcription factors can regulate several growth and development processes. However, there is little research on whether BBX is involved in flower formation and floral organ development of pineapple. In this study, *AcBBX5*, a BBX family gene with two conserved B-box domains, was identified from pineapple. Subcellular localization analysis showed that AcBBX5 was located in the nucleus. Transactivation analysis indicated that AcBBX5 had no significant toxic effects on the yeast system and presented transcriptional activation activity in yeast. Overexpression of *AcBBX5* delayed flowering time and enlarged flower morphology in Arabidopsis. Meanwhile, the expression levels of *AtFT*, *AtSOC1*, *AtFUL* and *AtSEP3* were decreased, and the transcription levels of *AtFLC* and *AtSVP* were increased in *AcBBX5*-overexpressing Arabidopsis, which might lead to delayed flowering of transgenic plants. Furthermore, transcriptome data and QRT-PCR results showed that *AcBBX5* was expressed in all floral organs, with the high expression levels in stamens, ovaries and petals. Yeast one-hybrid and dual luciferase assay results showed that AcBBX5 bound to *AcFT* promoter and inhibited *AcFT* gene expression. In conclusion, *AcBBX5* was involved in flower bud differentiation and floral organ development, which provides an important reference for studying the functions of BBX and the molecular regulation of flower.

## Introduction

Flowering, the symbol of vegetative development to reproductive development is an important factor to ensure the success of plant reproduction (Khan et al., 2014). The transition from vegetative stage to reproductive stage is precisely regulated by external environmental signals and internal developmental states (Albani and Coupland, 2010; Wang et al., 2021). Appropriate flowering time is crucial for the crop yield and quality (Roux et al., 2006). Flowering studies are the most extensive in Arabidopsis, and mainly involved photoperiod, vernalization, gibberellin, autonomic, ambient temperature and age-related pathways (Albani and Coupland, 2010; He, 2012; Cheng et al., 2017). Flowering is a very complex physiological process, which is regulated and coordinated by multiple genes (Liljegren et al., 1999).

*BBX* transcription factors have a wide range of functions and play important roles in flower initiation, light morphogenesis, anthocyanin synthesis and abiotic stress tolerance (Almada et al., 2009; An et al., 2020; Li et al., 2021). For example, *CmBBX24* regulates flowering time and tolerance to freezing and drought stress in chrysanthemum by regulating GA biosynthesis (Yang et al., 2014). AtBBX20 interacts with HY5 to activate the gene expression and promote light morphogenesis (Wei et al., 2016). *MdBBX22* and *MdBBX33* in apple are involved in MDHY5-mediated signal transduction and regulate anthocyanin accumulation (An et al., 2019; Plunkett et al., 2019). CONSTANS (CO) in Arabidopsis is the first BBX protein to be identified in plants. *Flowering locus T* (*FT*) genes receive signals from the photoperiodic regulatory center CO under long day conditions, which drives the transition from vegetative to reproductive growth in Arabidopsis (Putterill et al., 1995). However, CO could also inhibit *FT*-induced flowering by affecting *TERMINAL FLOWER1* (*TFL1*) expression under short-day conditions (Luccioni et al., 2019). Interestingly, in rice, *Hd1* is a CO homolog that promotes flowering under short-day conditions but inhibits flowering under long-day conditions (Yano et al., 2000; Kojima et al., 2002; Hayama et al., 2003). In flower formation pathway, BBX protein is strongly conserved among different plants. In addition to CO, other BBX proteins are also involved in the regulation of flower formation in plants. In Arabidopsis, both *AtBBX6* and *AtBBX24* are positive regulators of flower formation (Hassidim et al., 2009; Li et al., 2014), while *AtBBX4*, *AtBBX7* and *AtBBX32* delay flower formation (Cheng and Wang, 2005; Tripathi et al., 2017). *OsCOL4*, *OsBBX14* and *OsCOL9* in rice delayed the heading through repressing the Ehd1 pathway under SD and LD conditions (Lee et al., 2010; Bai et al., 2016; Liu et al., 2016). *BvCOL1* in sugar beet also causes early flowering under LD conditions (Chia et al., 2008).

Pineapple is one of the world’s famous tropical fruits. Spraying ethephon to induce flower formation is currently the most widely used method in pineapple production (Min and Bartholomew, 1997). At present, there are some excellent pineapple varieties that cannot be widely promoted in the market due to the difficulty in regulating the maturation time (Turnbull et al., 1999). Therefore, studying the molecular mechanism of flower formation induced by ethylene in pineapple can provide theoretical support for perinatal regulation and new variety cultivation. In recent years, several genes regulating flowering such as *AcERS* and *AcETR* (Li et al., 2016), *AcERF* (Zhang et al., 2021a), *AcTrihelix* (Wang et al., 2022), *AcBBX* (Ouyang et al., 2022) have been isolated from pineapple, but however, the research on its functional mechanism is still not in-depth.

BBX genes are involved in the determination of flowering time through photoperiod, and there are few studies on flower formation in response to the hormone regulation (Putterill et al., 1995; Yano et al., 2000). Previous studies have found that *AcBBX5* was found in the pineapple BBX family study to have an expression peak at 12 h and 7 w after ethylene induced flowering of pineapple. *AcBBX5* may be involved in ethylene induced flower formation and flower morphogenesis in pineapple (Ouyang et al., 2022). Here, *AcBBX5* was identified from the BBX family analysis of pineapple, and found it may regulate flower formation of pineapple. The sequence characteristics and expression characteristics of AcBBX5 were analyzed by bioinformatics, subcellular localization and transcriptional activation assay. *AcBBX5* overexpression, expression characteristics and regulatory mechanism were further analyzed. All these data lay a foundation for the further study of floral formation regulation network in pineapple.

## Materials and methods

### Plant materials and treatments

*Ananas comosus* L. cv. Comte de Paris was used as experimental material, and grown in pineapple resource nursery, Zhanjiang, China. The uniform pineapple plants (15-month-old) were treated with 30 mL 200 mg/L ethephon to induce flowering, and the control group with the same amount of water instead. The flower organs including petals, ovary, stamens, sepals and styles were collected separately in pineapple. All samples were performed with three biological replications and immediately frozen in liquid nitrogen and then stored at −80 °C until further use.

### RNA extraction and RT-qPCR assay

Total RNA was extracted with Polysaccharide Polyphenol Plant RNA Extraction Kit (Huayueyang, China) according to the manufacturer’s instructions. After detecting the concentration and quality of RNA by NanoDrop™ One/OneC Spectrophotometer (Thermo Fisher Scientific, USA), reverse transcription was carried out through the Revert Aid First-Strand cDNA Synthesis Kit (Thermo Fisher Scientific, USA). RT-qPCR was performed with ChamQ Universal SYBR qPCR Master Mix (Vazyme, China) on LightCycler 480 II (Roche, Switzerland). *AcActin* was used as an internal reference gene of pineapple. The reaction conditions for RT-qPCR consisted of predegeneration (95°C for 2 min), circular reaction (95 °C for 10 s, 58 °C for 30 s, 40 cycles) and Dissociation curve (95 °C for 15 s, 60 °C for 60 s, 95 °C for 15 s). All experiments incorporated three biological samples and three technical replicates. The relative expression levels of each gene were evaluated by using the 2^−ΔΔCt^ method (Rao et al., 2013).

### Bioinformatics analysis

The AcBBX5 protein sequence of pineapple was obtained from pineapple Genome Database (http://pineapple.zhangjisenlab.cn/pineapple/html/index.html) (Xu et al., 2018). The protein sequences of other species were all derived from NCBI (National Center for Biotechnology Information). MEME online site was used to analyze the conserved motifs (Bailey et al., 2009). The phylogenetic tree was constructed by neighbor-joining (NJ) method in MEGA 6.0 software, and the bootstrap value was set at 1000 replications (Hall, 2013).

### Subcellular localization and transcriptional activities

The coding sequence of *AcBBX5* was introduced into the pCAMBIA2300-GFP vector digested with *Kpn* I and *Xba* I restriction enzymes to generate the construct 35S::*AcBBX5*-GFP. After sequencing correctly, it was transformed into *Agrobacterium tumefaciens* strain GV3101. The epidermal cell transformation of tobacco leaves was injected with the *A. tumefaciens* carrying out with the recombinant vector. After incubation in the dark for 12 h at 25 °C, the tobaccos were transferred to normal growth for 24–36 h. The fluorescence signal was observed by a confocal scanning microscope Ax-io-Imager_LSM-800 (Zeiss, Germany) under excitation of 488 nm.

A yeast assay system was used to examine the transcriptional activity of *AcBBX5*. *Nde* I and *Sal* I were selected as restriction sites, and the coding sequences of *AcBBX5* were inserted into the bait vector pGBKT7 by homologous recombination. Following the manufacturer’s protocol, the recombinant vectors were transferred into AH109 strain yeast and cultured on SD/-Trp medium at 30 °C for 2–3 days. Single colonies were selected for amplification culture and then transferred to SD/-Trp and SD/-Trp/-His/-Ade solid media for further culture. Three days later, X-*α* -Gal was added to observe whether the colony was blue.

### Transformation and screening of AcBBX5 transgenic plants

*Arabidopsis thaliana* (ecotype Colombia) grown under conditions of 21 ± 1°C (day/night, 16/8 h) and used for heterologous transformation in this study. pCAMBIA2300-GFP-*AcBBX5* vectors (the recombinant vectors were used for subcellular localization) were used to transform into *Arabidopsis via* the floral dip method (Clough and Bent, 1998). Empty vectors were transformed as control. The seeds of the T0 generation were harvested and screened on plates containing MS medium with 50 mg/L kanamycin sulfate and then further verified the transgenic plants by PCR amplification. The T3 generation transgenic *Arabidopsis* lines were used for subsequent phenotype observation and functional analysis. To detect the expression levels of the *AcBBX5* and some flowering-related genes in transgenic and control plants, 28-day-old seedlings of both transgenic and control *Arabidopsis* plants were collected for qRT-PCR analysis.

## Results

### Characterization analysis of AcBBX5

**Figure 1:**
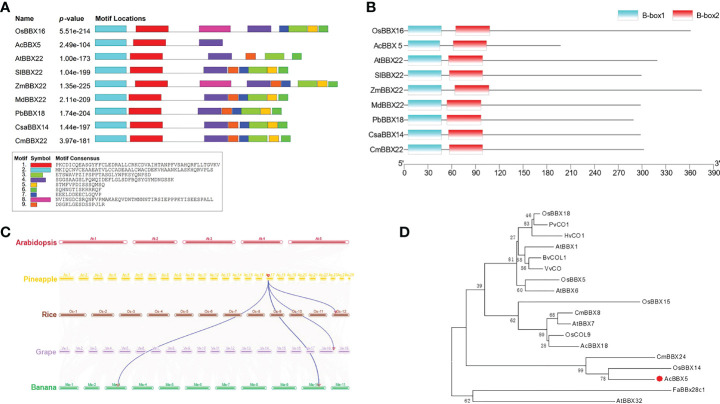
Characterization of AcBBX5 sequence in pineapple. **(A)** Motif analysis of AcBBX5 proteins. Different colored boxes represent the different types of motifs. **(B)** Domain analysis of AcBBX5 proteins. **(C)** Syntenic relationships of AcBBX5 in pineapple with rice, grape and banana. **(D)** Phylogenetic analyses of AcBBX5. Phylogenetic tree based on BBX proteins involved in flower formation regulation.

To investigate the function of the AcBBX5 protein, we first identified and cloned AcBBX5 from the ‘Paris’ pineapple by RT-PCR. The coding sequence of AcBBX5 was 603 bp in length, encoding 197 amino acid residues. In order to better understand the properties of AcBBX5, the amino acid sequence of AcBBX5 was analyzed. The results showed that AcBBX5 and its homologous proteins in other species all contained two B-box conserved domains, and contained motif 1, motif 2 and motif 4 in conserved motif analysis (**Figure 1A**). However, compared with homologous protein sequences of other species, AcBBX5 sequence was significantly shorter and lacks motif 3, which was common in other sequences. Interestingly, the positions of motifs 1 and 2 in the sequence almost overlapped with those of the two B-box conserved domains, and both motifs 1 and 2 might represent B-box conserved domains. Furthermore, multiple sequence alignments with homologous amino acid sequences in *Arabidopsis*, rice, pear, cucumber and tomato revealed that the amino acid sequence similarity between AcBBX5 and homologous proteins in other species were 24.8% (AtBBX22), 24.53% (CsaBBX14), 24.27% (OsBBX16), 22.93% (PbBBX18), and 25.60% (SlBBX22), respectively (**Figure S1**). The low sequence similarity is probably due to the absence of a fragment at the C-terminal of AcBBX5 relative to homologous sequences of other species.

The function of BBX protein has been studied in many plants. Collinearity analysis of pineapple with rice, grape, and banana revealed that pineapple AcBBX5 has one homologous gene pair in rice and banana, but two in grape (**Figure 1**). In order to investigate the role of AcBBX5 protein in flower formation, 18 BBX proteins that have been confirmed to be involved in flower formation regulation were selected to construct phylogenetic tree (**Figure 1** and **Table S1**). Among them, there were six BBX proteins promoting flowering (BvCOL1, AcBBX18, HvCO1, VvCO, AtBBX6 and CmBBX8) and 9 delayed flowering proteins (AtBBX32, AtBBX7, BvCOL1, CmBBX24, FaBBx28c1, OsBBX14, OsBBX15, OsCOL9, OsBBX27 and PvCO1). AtBBX1 and OsBBX18 are genes that can both promote and inhibit flower formation. Phylogenetic tree analysis showed that AcBBX5 and delayed flowering proteins (CmBBX24 and OsBBX14) clustered in the same branch, suggesting that their functions may be similar. AcBBX5 may have the function of delaying flower formation as CmBBX24 and OsBBX14.

### Subcellular localization and transcriptional activation activity of AcBBX5

**Figure 2:**
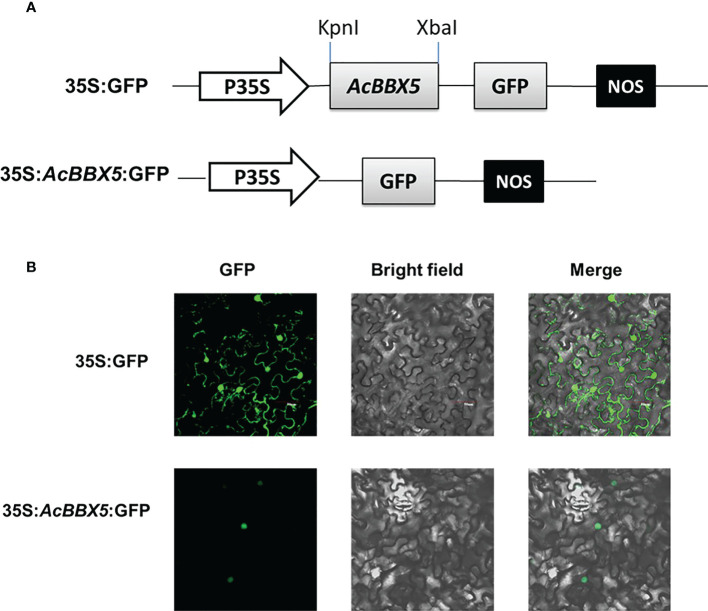
Subcellular localization of AcBBX5 in tobacco leaf cells. **(A)** Vectors for *AcBBX5* subcellular localization analysis. **(B)** AcBBX5 was localized in tobacco leaf epidermal cells. 2300-GFP and 2300-*AcBBX5*-GFP plasmids were transformed into tobacco. The dark field, bright field and merge field were shown in the left, middle and right panels, respectively.

Subcellular localization information is of great significance to our understanding of protein function. To investigate the subcellular localization of the AcBBX5 protein, 35S::*AcBBX5*-GFP protein was transiently expressed in *Nicotiana benthamiana* leaves (**Figure 2**). Under confocal microscope, the GFP fluorescence of the control vector was distributed in the nucleus and the cell membrane, while the fluorescence signals of 35S::*AcBBX5*-GFP were detected in only the nucleus. The results indicate that AcBBX5 localizes in the nucleus, and may act as a transcription factor in the nucleus to participate in the transcriptional regulation of other gene expression.

**Figure 3:**
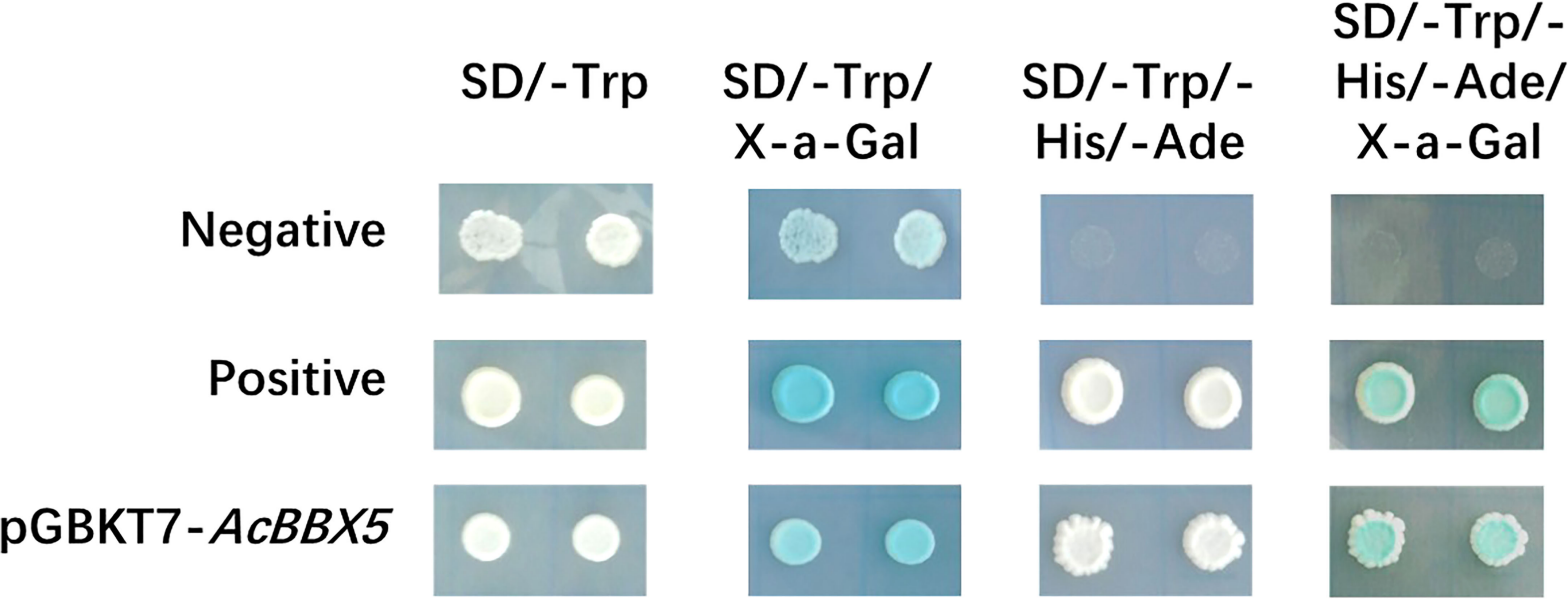
Transcriptional activation activity analysis of AcBBX5 in yeast. The empty vector pGBKT7 was transformed into yeast as the negative control; pGADT7-T and pGBKT7-53 were co-transformed into yeast was used as a positive control.

The detection of transcriptional activation and yeast toxicity provides a foundation for further exploring the mechanism of *AcBBX5*. The complete coding region of AcBBX5 was fused to the GAL4-binding domain in the pGBKT7 vector, and expressed in the yeast strain Y2H. The pGADT7-T and pGBKT7-53 vectors served as the positive control, while the empty pGBKT7 vector was negative control. As shown in **Figure 3**, the positive control strains and the yeast cells with the pGBKT7-*AcBBX5* vectors grew extremely well on both SD/-Trp and SD/-Trp/-His/-Ade medium, and could turn blue on medium coated with X-α-gal, whereas those negative control strains were unable to grow on SD/-Trp/-His/-Ade medium, which confirms that AcBBX5 has no significant toxic effects on the yeast system and exhibited transcriptional activation activity in yeast.

### Overexpression of *AcBBX5* significantly delays the flowering time in Arabidopsis

**Figure 4:**
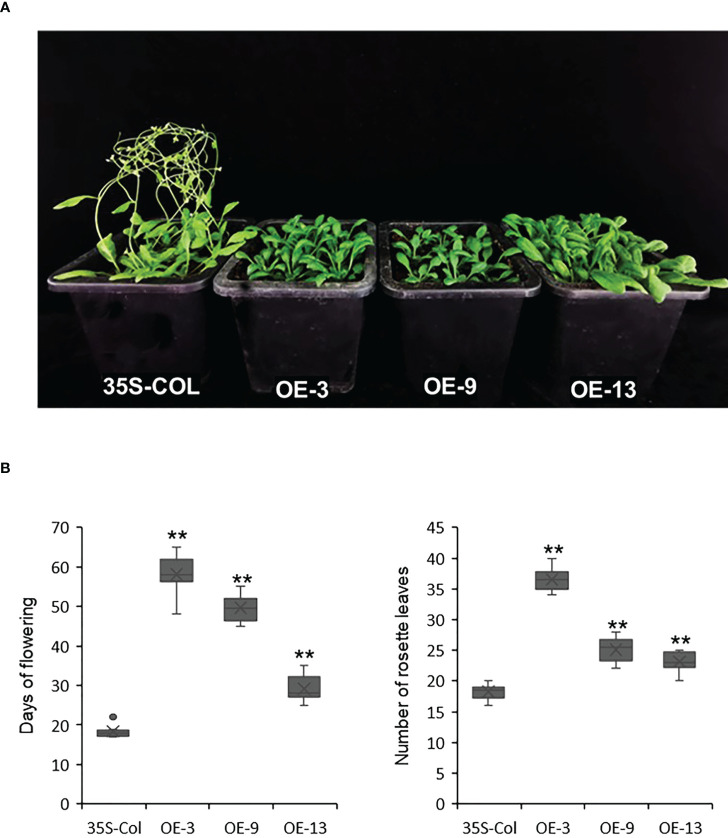
Ectopic expression of AcBBX5 in Arabidopsis. **(A)** Phenotypes analysis of T3 transgenic Arabidopsis with *AcBBX5*. **(B)** Days to flowering and number of rosette leaves at flowering in transgenic plants (n=8). Asterisks indicate significant differences. (*P < 0.1, **P < 0.05, based on Student’s t-test).

**Figure 5:**
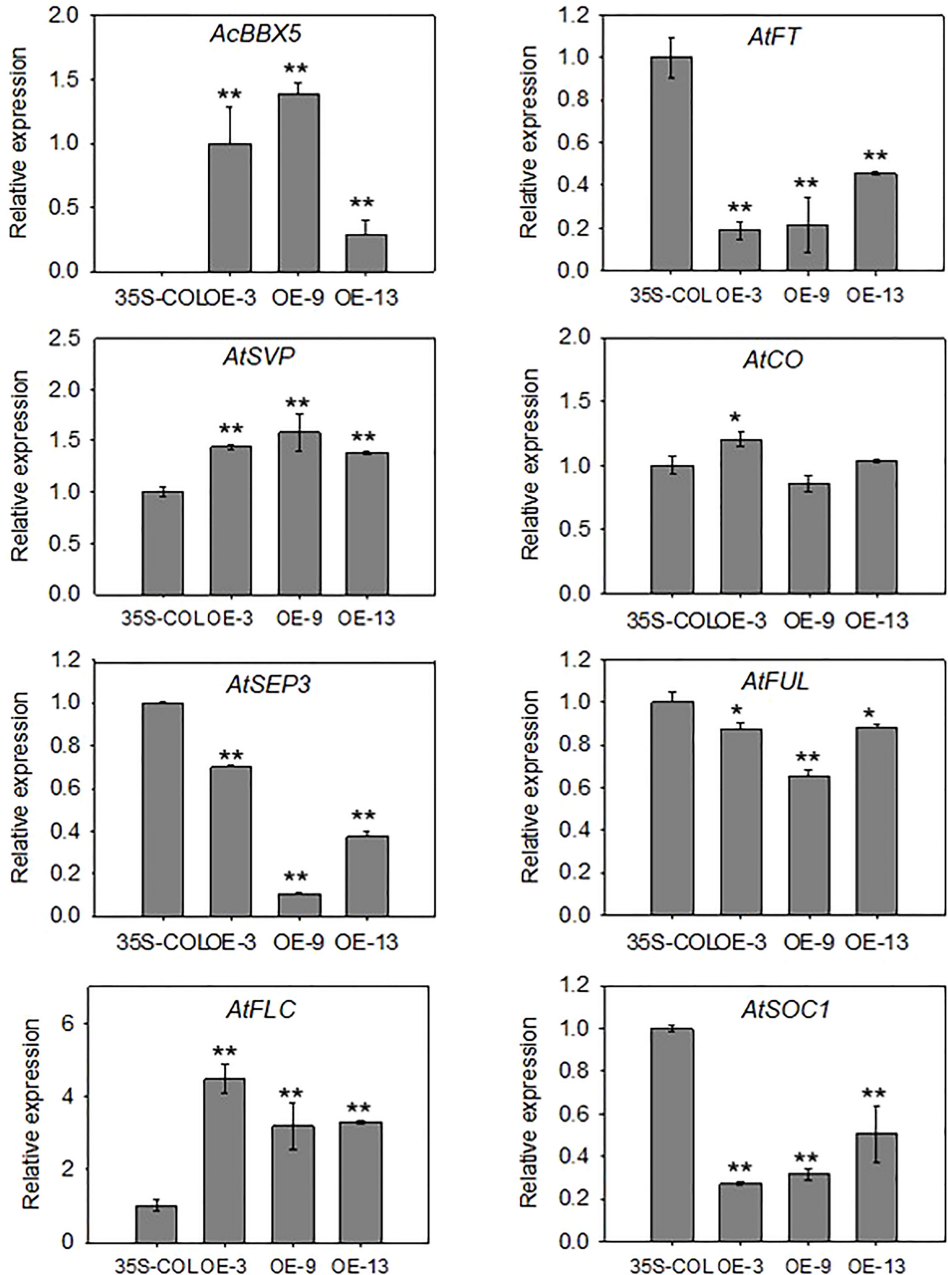
Expression analysis in transgenic Arabidopsis plants. RT-qPCR analysis of *AcBBX5* and genes involved in flowering, including *AtSEP3*, *AtSVP*, *AtCO*, *AtFLC*, *AtSOC1*, *AtFUL* and *AtFT*, in transgenic Arabidopsis plants (n=3). Asterisks indicate significant differences. (*P < 0.1, **P < 0.05, based on Student’s t-test).

Previous studies have found that *AcBBX5* had a high expression peak at 7 w after ethylene induction, which was the stage of floret development (Ouyang et al., 2022). To evaluate whether *AcBBX5* is involved in flower formation, overexpressed Arabidopsis transgenic lines of *AcBBX5* were constructed. Transgenic plants carrying pCAMBIA2300-GFP empty vector were used as negative control. Three highly expressed lines were selected from T3 homozygous positive transgenic lines (OE-3, OE-9 and OE-13) by PCR amplification and QRT-PCR to study their characteristics (**Figure 4A**). All three *AcBBX5* transgenic lines flowered significantly later than control Arabidopsis, and rosette numbers were significantly higher than those of control. In the long day (16 h light/8 h dark) condition, the control plants with about 18 rosette leaves began to blossom about 18 days after transplanting. The overexpressed OE-3, OE-9 and OE-13 bloomed at about 58, 50 and 29 days after transplanting, and the number of rosette leaves was about 37, 25 and 23, respectively (**Figure 4B**). We also detected the transcript levels of *AcBBX5* in T3 lines of OE-3, OE-9 and OE-13 Arabidopsis respectively, and found that expression levels of *AcBBX5* in different transgenic lines were significantly increased. Interestingly, the flowering time was negatively associated with the expression of *AcBBX5* in transgenic Arabidopsis plants (**Figure 5**). These results indicated that overexpression of AcBBX5 in Arabidopsis leaded to a serious delay in flowering time.

In order to better understand the molecular mechanism of *AcBBX5* involved in flowering time regulation, the cDNAs of *AcBBX5* transgenic Arabidopsis lines as template were used to analyze the genes involved in flower regulation in Arabidopsis by QRT-PCR (**Figure 5**). Seven genes involved in the determination of flowering time and flower development were searched from the Arabidopsis flowering database (http://www.phytosystems.ulg.ac.be/florid/). Compared with the control plants, the expression levels of *FT*, *SUPPRESSOR OF OVEREXPRESSION OF CO1* (*SOC1*), *FRUITFULL* (*FUL*) and *SEPALLATA3* (*SEP3*) genes promoting flower formation in Arabidopsis were inhibited in overexpressed lines, while the expression levels of *FLOWERING LOCUS C* (*FLC*) and *SHORT VEGETATIVE PHASE* (*SVP*) genes negatively regulating flower formation were significantly increased in transgenic Arabidopsis plants. These results further confirmed that *AcBBX5* repressed flower formation in Arabidopsis by regulating the expression of other floral genes and this might suggest the possibility that *AcBBX5* represses flowering in Pineapple.

### Overexpression of *AcBBX5* can enlarge the floral morphology of the transgenic Arabidopsis

**Figure 6:**
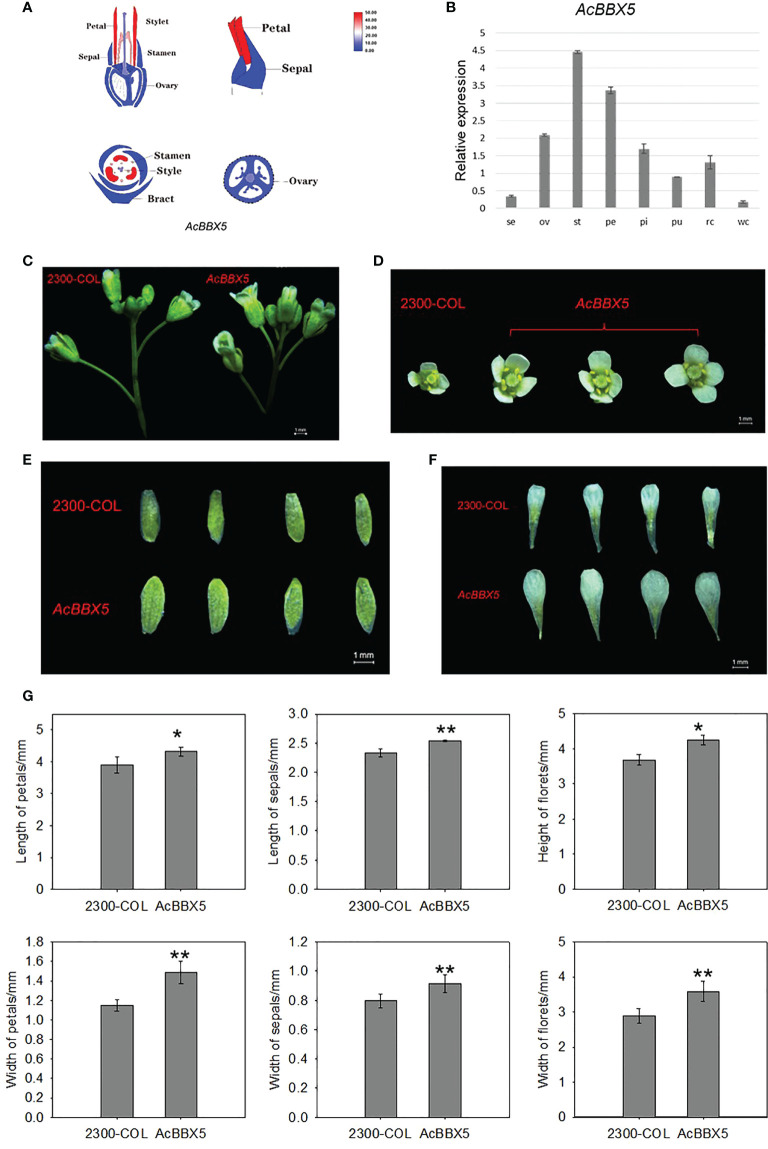
Phenotypes and expression characteristics of floral organs in transgenic Arabidopsis. **(A)** Transcriptome data. **(B)** RT-QPCR. Se, Sepal; Pe, Petal; St, Stamen; Ov, ovary; St, Style; Pu, Fruit; Rc, Root; and Wc, Leaf. **(C–F)** Phenotypes characteristics of floral organs. **(G)** Statistical analysis of floret height, floret width, petal length, petal width, sepal length and sepal width of AcBBX5 transgenic lines and control lines (n=3). Asterisks indicate significant differences. (*P < 0.1, **P < 0.05, based on Student’s t-test).

In addition to delay flowering, florets were also larger in AcBBX5 transgenic lines compared to control lines at full flowering (**Figure 6**). To test whether AcBBX5 is involved in floral organ development, transcriptome data and QRT-PCR were used to analyze floral organs of pineapple, including sepal, ovary, stamen, petal and pistil. The results demonstrated that AcBBX5 was specifically expressed in the floral organ with higher expression levels in stamen and petals. The results showed that AcBBX5 was specifically expressed in floral organs, with higher expression levels in stamens and petals. The phenotypic characteristics of AcBBX5 were evaluated by comparing the sepal and petal sizes of AcBBX5 and control (35S-COL) flowers. Compared with the control plants, the width of petals and sepals in the transgenic plants was larger, suggesting that AcBBX5 was also involved in floral organ development.

### *AcBBX5* regulates the expression of *AcFT* in pineapple

**Figure 7:**
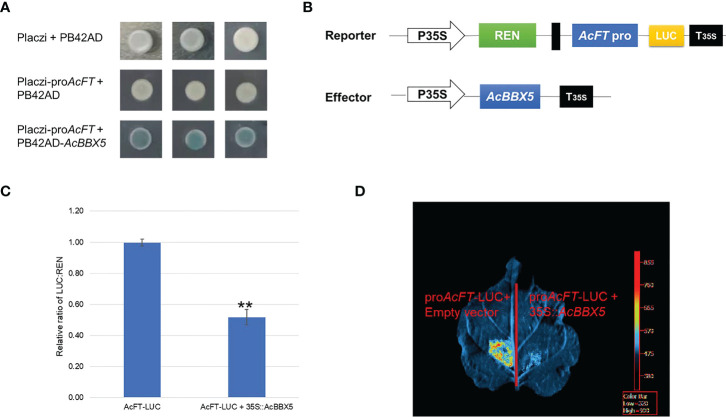
*AcBBX5* binds to *AcFT* promoter and negatively regulates its expression. **(A)** Yeast one-hybrid assay to test whether *AcBBX5* could directly bind to the promoters of *AcFT* **(B)** Schematic diagram of effector and reporter structures used for dual luciferase assay. LUC, firefly luciferase. REN, Renilla luciferase. P35S and T35S, the promoter and terminator of CaMV35S, respectively. **(C)** The comparison of luciferase activity (n=3). Asterisks indicate significant differences. (*P < 0.1, **P < 0.05, based on Student’s t-test). **(D)** Representative bioluminescence image of AcBBX5 activation on the *AcFT* promoter in tobacco leaves.

To further identify the genes directly regulated by *AcBBX5* in pineapple flower formation, yeast one-hybrid assay was performed to determine whether *AcBBX5* could bind to the promoter of *AcFT*. PB42AD-*AcBBX5* and Placzi-pro*AcFT* vectors were combined to transform yeast strain EGY48. PB42AD/Placzi- pro*AcFT* and Placzi/PB42AD-*AcBBX5* were used as negative control. The transformed yeast showed blue color in SD/Trp-Ura- medium containing X-Gal, but the negative control did not. This result indicated that AcBBX5 could bind to the promoter of *AcFT* (**Figure 7A**). In addition, the reporter (pGreenII0800-LUC-pro*AcFT*) and effector plasmid (35S:AcBBX5) were constructed and a dual luciferase (LUC) assay was performed in N. benthamiana leaf cells to examine whether *AcBBX5* could activate the *AcFT* promoter. As expected, in the LUC reporter assays, the fluorescence signal was significantly attenuated in sites infected by *AcBBX5* and *AcFT* promoters compared with the negative control, and the activity of the promoter expressed by the LUC/REN ratio was significantly reduced relative to the control (**Figure 7B**). In conclusion, AcBBX5 can bind to the *AcFT* promoter and inhibit its expression, thereby inhibiting the flower formation of pineapple.

## Discussion

The BBX gene family is widely involved in plant growth and development and response to the environment. *AcBBX5* is a member of the pineapple *AcBBX* family (Ouyang et al., 2022). Here, conserved domain analysis confirmed that AcBBX5 and other orthologous proteins contain two conserved B-box domains at the N-terminus (**Figure 1A**). Motif analysis identified two conserved B-box domains containing a different conserved motif (**Figure 1B**). Based on the difference in amino acid sequence identity of the b-box motif and the specificity of zinc binding amino acid residues, it is divided into two types: B-box 1 and B-box 2 (Crocco and Botto, 2013). Plant B-box domains can form heterodimers within the BBX protein family or with other proteins, and play an important role in mediating protein interactions and regulating the gene expression (Gangappa et al., 2013). For example, PpBBX18 in pear forms a heterodimer with PpHY5 *via* two b-box domains, thereby inducing *PpMYB10* transcription and regulating anthocyanin biosynthesis (Bai et al., 2019). Subcellular localization analysis showed that AcBBX5 protein was located in the nucleus of epidermal cells of *Nicotiana benthamiana* leaves (**Figure 2**) and had transcriptional activation activity in yeast (**Figure 3**), suggesting that *AcBBX5* gene has general characteristics of transcription factor and may be involved in the transcription level of other genes. However, **Figure S1** shows that the sequence length of AcBBX5 was significantly shorter than the others. In addition, except for AcBBX5, which has only 3 conserved motifs, other homologous proteins have at least 6 conserved motifs (**Figure 1A**). These imply that the function of AcBBX5 may be different from that of homologous genes in other species.

BBX genes are involved in the determination of flowering time (Putterill et al., 1995; Yano et al., 2000). *AtBBX24*, *CmBBX8* and *HvCO1* are the *BBX* genes that have been shown to promote flower formation. *AtBBX24* overexpression not only reduces the expression level of *FLC*, but also activates *FT* and *SOC1* expression, which leads to early flowering in Arabidopsis under long- and short-day conditions (Li et al., 2014). In summer chrysanthemum, overexpression of *CmBBX8* accelerated flowering under long- and short-day conditions. *CmFTL1* can act as floral inducer in long day conditions, and *CmBBX8* promotes flowering through binding with CORE element (CCACA) of the *CmFTL1* promoter (Wang et al., 2020). In barley, *HvCO1* overexpression up-regulated *HvFT1*, which may promote flowering by activating HvFT1. But it has no promoting effect on Arabidopsis thaliana (Armstead et al., 2005). In this study, overexpression of AcBBX5 gene in Arabidopsis delayed flowering time (**Figure 4**). BBX has also been reported to inhibit floral formation in other species. In rice, OsBBX14 delays heading date *via* different ways under long and short-day conditions. OsBBX14 delays heading date by promoting the expression of Hd1 under long day conditions. However, under short day conditions, it acts as a repressor of Ehd1 to delay heading date (Bai et al., 2016). *CmBBX24* may inhibit flowering in *Chrysanthemum Morifolium* by negatively regulating the expression of GA biosynthetic genes (*GA20ox* and *GA3ox*) and photoperiodic flowering pathway genes (*GI*, *PRR5*, *CO*, *FT* and *SOC1*) (Yang et al., 2014). *OsBBX14* and *CmBBX24*, which inhibit flower-forming genes, cluster on the same branch of the evolutionary tree with *AcBBX5* (**Figure 1D**), suggesting that genes of the same classification or more closely related genes may have similar functions.

Molecular studies on flowering have focused on *FT*, *FLC*, *CO* and *SOC1* genes. In addition to *AtBBX24* (Li et al., 2014), which has been shown to regulate *FLC*, *FT* and *SOC1*, *AtBBX7*/*AtCOL9* has been reported to inhibit *CO* and *FT* expression and delay floral transition in Arabidopsis (Cheng and Wang, 2005). The heterodimerization between *BBX28* and *CO* affects the activation of *FT* transcription by CO, which negatively regulates Arabidopsis flower formation (Liu et al., 2020). In this study, the relative expression levels of positive regulators of flower formation (*FT*, *SEP3*, *SOC1* and *FUL*) were inhibited in Arabidopsis transgenic lines, while negative regulators of flower formation (*FLC* and *SVP*) were higher than control lines (**Figure 5**). This further confirmed that *AcBBX5* overexpression inhibited flowering in Arabidopsis. However, the relative expression of *CO* in the Arabidopsis transgenic lines was almost no different from that in the control, indicating that *AcBBX5* does not negatively regulate flower formation by inhibiting *CO* expression.

Yeast one-hybrid and dual luciferase assay results found that AcBBX5 is transcriptional activator (**Figure 3**), but it can bind to the promoter of *FT* gene and inhibit its expression (**Figure 7**). Similarly, previous studies on transcription factors in other species have also confirmed that transcription factors with transcriptional activation activity can also act as repressors under certain conditions. In grape hyacinth, MaBBX51 interfered with the binding of MaHY5 to the promoters of *MaMybA* and *MaDFR*, thereby inhibiting anthocyanin biosynthesis (Zhang et al., 2022). VvMYB30 in grapevine competes with activator VvMYB14 to bind to common binding sites in *VvSTS15/21* promoter, controls stilbene biosynthesis in grapevine (Mu et al., 2022). In grape, VvMYB30 competed with the activator VvMYB14 to bind to characteristic binding sites in the *VvSTS15/21* promoter to effect stilbene biosynthesis (Mu et al., 2022). In Arabidopsis, AtBBX24 inhibited anthocyanin accumulation by interfering with the binding of *HY5* to the promoter of related genes in anthocyanin biosynthesis (Job et al., 2018). AtBBX19 negatively regulated flowering time by interfering with CO binding to *FT* through physical interaction with CO proteins (Wang et al., 2014). In view of this, BBX5 may act as a weak activator, which binds to the *FT* promoter of the target gene and occupies a limited binding site to block the binding of some strong activators, thus inhibiting the expression of *FT* gene. This speculation still needs further systematic experimental verification.

A trait is regulated by multiple genes, and a gene may be involved in the regulation of multiple traits. Here, *AcBBX5* may not only regulate the flower formation of pineapple (**Figure 4**), but also promote the growth and development of floral organs (**Figure 6**). *BBX* gene is one of the many genes involved in flower organ growth and development. However, there are few studies on the BBX gene in flower organs. In rose, *RhBBX28* is a key player in regulating petal senescence, and overexpression of *RhBBX28* produces smaller flowers than WT (Zhang et al., 2021b). In the present study, *AcBBX5* overexpression also showed differences in flower size in Arabidopsis. But in contrast to *RhBBX28*, overexpression of *AcBBX5* produced larger flowers than the control (**Figure 6**). Transcriptome and quantitative data showed that *AcBBX5* was highly expressed in petals, suggesting that *AcBBX5* may play a role in petal development. Further studies and sufficient experimental evidence are needed to prove the specific regulatory mechanism.

## Conclusions

In this study, *AcBBX5*, a member of the BBX family, is identified as a negative regulator of floral formation in Arabidopsis and may be involved in floral organ development. Subcellular localization and transcriptional activation analysis showed that AcBBX5 was located in the nucleus and had transcriptional activation potential. The relative expressions of FT, *SOC1*, *FUL* and *SEP3* were decreased, and *FLC* and *SVP* were increased in *AcBBX5*-overexpressing Arabidopsis. In addition, AcBBX5 was expressed in different floral organs of pineapple, but highly expressed in stamens, ovary and petals. Yeast one-hybrid and a dual luciferase assay results confirmed that AcBBX5 bound to *AcFT* promoter in pineapple and inhibited the expression of *AcFT* gene. These data will enrich the known regulatory network of BBX in different plants and provide information on the regulation of flowering and floral organ development in pineapple.

## Data availability statement

The original contributions presented in the study are included in the article/**Supplementary Material**. Further inquiries can be directed to the corresponding author.

## Author contributions

YO and HZ wrote the manuscript and completed the experiments. XZ and HZ designed the experiment and contributed to data. YH took charge the experimental materials treatment and collection. YW investigated the data analyze of the study. YO and CW carried out RNA extraction and QRTPCR verification. XZ and ZL constructed the vectors and completed the transgenic experiments. HZ and XZ conceived the study. All authors contributed to the article and approved the submitted version.

## Funding

The project was funded by the National Natural Science Fund of China (31872079 and 32160687), the National Key R&D Program of China (2019YFD1001105 and 2018YFD1000504), the Natural Science Foundation of Hainan Province (321RC467 and 322MS013), the major science and technology project of Hainan Province (ZDKJ2021014), and the Scientific Research Start-up Fund Project of Hainan University (KYQD-ZR-20090).

## Conflict of interest

The authors declare that the research was conducted in the absence of any commercial or financial relationships that could be construed as a potential conflict of interest.

## Publisher’s note

All claims expressed in this article are solely those of the authors and do not necessarily represent those of their affiliated organizations, or those of the publisher, the editors and the reviewers. Any product that may be evaluated in this article, or claim that may be made by its manufacturer, is not guaranteed or endorsed by the publisher.

## Supplementary material

Supplementary Figure 1 Protein alignment of AcBBX5 and its homologs from other species.
