# Supplementary material for: AcBBX5, a B-box transcription factor from pineapple, regulates flowering time and floral organ development in plants
Source: Front Plant Sci. 2022 Nov 24;13:1060276. doi: 10.3389/fpls.2022.1060276 (PMC9729951; doi:10.3389/fpls.2022.1060276)
Supplement: Supplementary file 1 [file Table_1.docx]

**Table S1.** The protein information

| **Protein** | **Length** | **species** | **Source** |
| --- | --- | --- | --- |
| AtBBX1 | 373 | *Arabidopsis thaliana* | Putterill et al., 1995 |
| AtBBX6 | 355 | *Arabidopsis thaliana* | Hassidim et al., 2009 |
| AtBBX7 | 372 | *Arabidopsis thaliana* | Cheng and Wang, 2005 |
| AtBBX22 | 319 | *Arabidopsis thaliana* | NP_001185428 |
| AtBBX32 | 225 | *Arabidopsis thaliana* | Tripathi et al., 2017 |
| BvCOL1 | 367 | *Beta vulgaris* | Chia et al., 2008 |
| CmBBX8 | 367 | *Chrysanthemum* | Wang et al., 2020 |
| CmBBX22 | 302 | *Chrysanthemum* | AMO42709.1 |
| CmBBX24 | 245 | *Chrysanthemum* | Yang et al., 2014 |
| CsaBBX14 | 298 | *Cucumis sativus* | XP_004137228.1 |
| FaBBx28c1 | 252 | *Fragaria x ananassa* | Ye et al., 2021 |
| HvCO1 | 383 | *Hordeum vulgare* | Armstead et al., 2005 |
| MdBBX22 | 298 | *Malus domestica Borkh* | XP_008348545.2 |
| OsBBX14 | 378 | *Oryza sativa* | Bai et al., 2016 |
| OsBBX15 | 224 | *Oryza sativa* | Zhang et al., 2015 |
| OsBBX16 | 361 | *Oryza sativa* | Huang et al., 2012 |
| OsCOL9 | 406 | *Oryza sativa* | Liu et al., 2016 |
| PbBBX18 | 289 | *Pyrus bretschneideri Rehd.* | Cao et al., 2017 |
| PvCO1 | 384 | *Ph. violascens* | Xiao et al., 2018 |
| SlBBX22 | 299 | *Solanum lycopersicum* | XP_004244294.1 |
| VvCO | 358 | Vitis vinifera | Almada et al., 2009 |
| ZmBBX22 | 375 | *Zea mays* | NP_001131712.1 |

**References**

Cao, Y., Han, Y., Meng, D., Li, D., Jiao, C., Jin, Q., et al. (2017). B-BOX genes: genome-wide identification, evolution and their contribution to pollen growth in pear (Pyrus bretschneideri Rehd.). *BMC plant biol.* 17, 156.

Huang, J., Zhao, X., Weng, X., Wang, L., and Xie, W. (2012). The rice b-box zinc finger gene family: genomic identification, characterization, expression profiling and diurnal analysis. *PloS One* 7, e48242.

Xiao, G., Li, B., Chen, H., Chen, W., Wang, Z., Mao, B. and et al. (2018). Overexpression of PvCO1, a bamboo CONSTANS-LIKE gene, delays flowering by reducing expression of the FT gene in transgenic Arabidopsis. *BMC Plant Biol.* 18, 232.

Ye, Y., Liu, Y., Li, X., Wang, G., Zhou, Q., Chen, Q., et al. (2021). An evolutionary analysis of B-Box transcription factors in strawberry reveals the role of FaBBx28c1 in the regulation of flowering time. *Int J Mol Sci.* 22, 11766.

Zhang, L., Li, Q., Dong, H., He, Q., Liang, L., Tan, C., et al. (2015). Three CCT domain-containing genes were identified to regulate heading date by candidate gene-based association mapping and transformation in rice. *Sci Rep.* 5, 7663.
