# Supplementary material for: AcBBX5, a B-box transcription factor from pineapple, regulates flowering time and floral organ development in plants
Source: Front Plant Sci. 2022 Nov 24;13:1060276. doi: 10.3389/fpls.2022.1060276 (PMC9729951; doi:10.3389/fpls.2022.1060276)
Supplement: Supplementary Figure 1 — Protein alignment of AcBBX5 and its homologs from other species. [file Image_1.pdf]

Figure S1

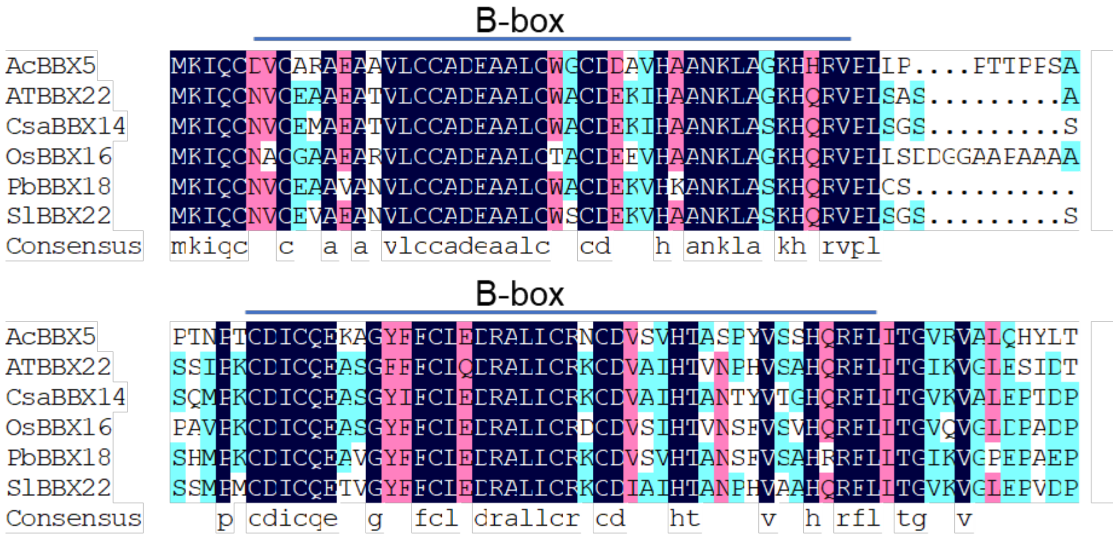
B-box
AcBBX5
ATBBX22
CsaBBX14
OsBBX16
PbBBX18
SlBBX22
Consensus
B-box
AcBBX5
ATBBX22
CsaBBX14
OsBBX16
PbBBX18
SlBBX22
Consensus
